# Rebalancing health investment across the life course: a high-value age segments framework for aging societies

**DOI:** 10.3389/fpubh.2026.1817328

**Published:** 2026-07-02

**Authors:** Jin Tian, Zhihua Zhang, Biao Xi

**Affiliations:** 1 Hospital Management Innovation Research Center, The First Hospital of Hebei Medical University, Shijiazhuang, China; 2 Medical Education Collaborative Innovation Center, Hebei Medical University, Shijiazhuang, China

**Keywords:** health expenditure prioritization, health system efficiency, high-value age segments, life-course health investment, population aging, preventive health strategy

## Abstract

Health systems in rapidly aging societies continue to direct a disproportionate share of expenditure toward reactive, late-life care, despite evidence that earlier and mid-life investment can generate substantial long-term population health returns. High-Value Age Segments (HVAS) are proposed as a life-course framework for prioritizing health investment according to differences in marginal returns across age groups. Three broad periods are identified: early life from the fetal stage to approximately age 25, mid-adulthood from ages 26 to 55, and later adulthood from age 56 onward. Early-life interventions are associated with durable gains in health and human development, mid-adulthood interventions with delayed chronic disease onset and preserved productivity, and later-life interventions with reduced morbidity and maintenance of functional capacity. Embedding HVAS within policy frameworks may support more efficient and sustainable allocation of health resources under demographic aging.

## 1 Introduction

The Western Pacific Region is undergoing an unprecedented demographic transition. More than 265 million people aged 65 years and over, representing approximately one third of the global older population, currently reside in this region, and the number is projected to double by 2050 (1). At the same time, fertility rates across East Asia have declined to historically low levels, including 0.73 in South Korea, 0.95 in Singapore, 1.01 in China, and 1.22 in Japan (2). Population aging and sustained fertility decline are reshaping age structures and dependency ratios, placing growing fiscal and operational pressure on health systems. Although this article focuses on the Western Pacific Region as defined by the World Health Organization, similar demographic and epidemiological patterns are evident in many other rapidly aging settings.

Health systems across the region remain predominantly oriented toward reactive care, responding to established disease and functional decline rather than investing earlier to preserve health. A substantial share of health expenditure is directed toward treatment and late-life care, while comparatively limited investment is directed toward maintaining health across the life course (3–5). Current allocation patterns provide little guidance on how limited health resources should be prioritized across different stages of life. Extending life expectancy without preserving functional capacity increases the likelihood of prolonged morbidity and dependency (6, 7). Evidence from multiple high-income countries shows that years lived with disability have risen alongside life expectancy, suggesting an expansion rather than compression of morbidity.

Existing policy frameworks have addressed important aspects of population aging, particularly functional ability and integrated care in later life (8). Yet they provide less guidance on how health investment should be timed across the full life course. Differences in the long-term return of health investment across age groups seldom shape real-world allocation decisions, even though the timing of intervention strongly influences downstream outcomes. Without an explicit age-stratified approach, health systems risk continuing to spend most heavily after disease and dependency have already become established.

To address this gap, this article proposes High-Value Age Segments as a life-course framework for identifying stages at which health investment is likely to generate greater population-level returns. By linking return-on-investment logic with life-course health strategy, the framework offers a structured basis for shifting health expenditure toward stages where earlier intervention can produce cumulative benefits. Its aim is not to reduce investment in older populations, but to make the timing of health investment a visible criterion in resource allocation for aging societies.

## 2 Conceptualizing high-value age segments (HVAS)

High-Value Age Segments are defined as periods within the life course during which marginal health investment yields disproportionately greater gains in healthy life expectancy and disability-adjusted life-years. In this formulation, value denotes population-level return on investment rather than the intrinsic worth of individuals at different ages. Life-course health theory and human capital models have established that health trajectories are cumulative and path-dependent (9, 10). Grossman's health capital model (10) and Heckman's analysis of declining returns to human capital investment with age (11) together provide a theoretical basis for expecting higher marginal returns in early and mid-life relative to later adulthood, a pattern further supported by developmental epidemiology including the developmental origins of health and disease (DOHaD) framework. However, existing frameworks provide limited operational guidance for allocating finite resources across age strata. The HVAS framework responds to this limitation by translating return-on-investment logic into actionable life-course priorities.

**Figure 1:**
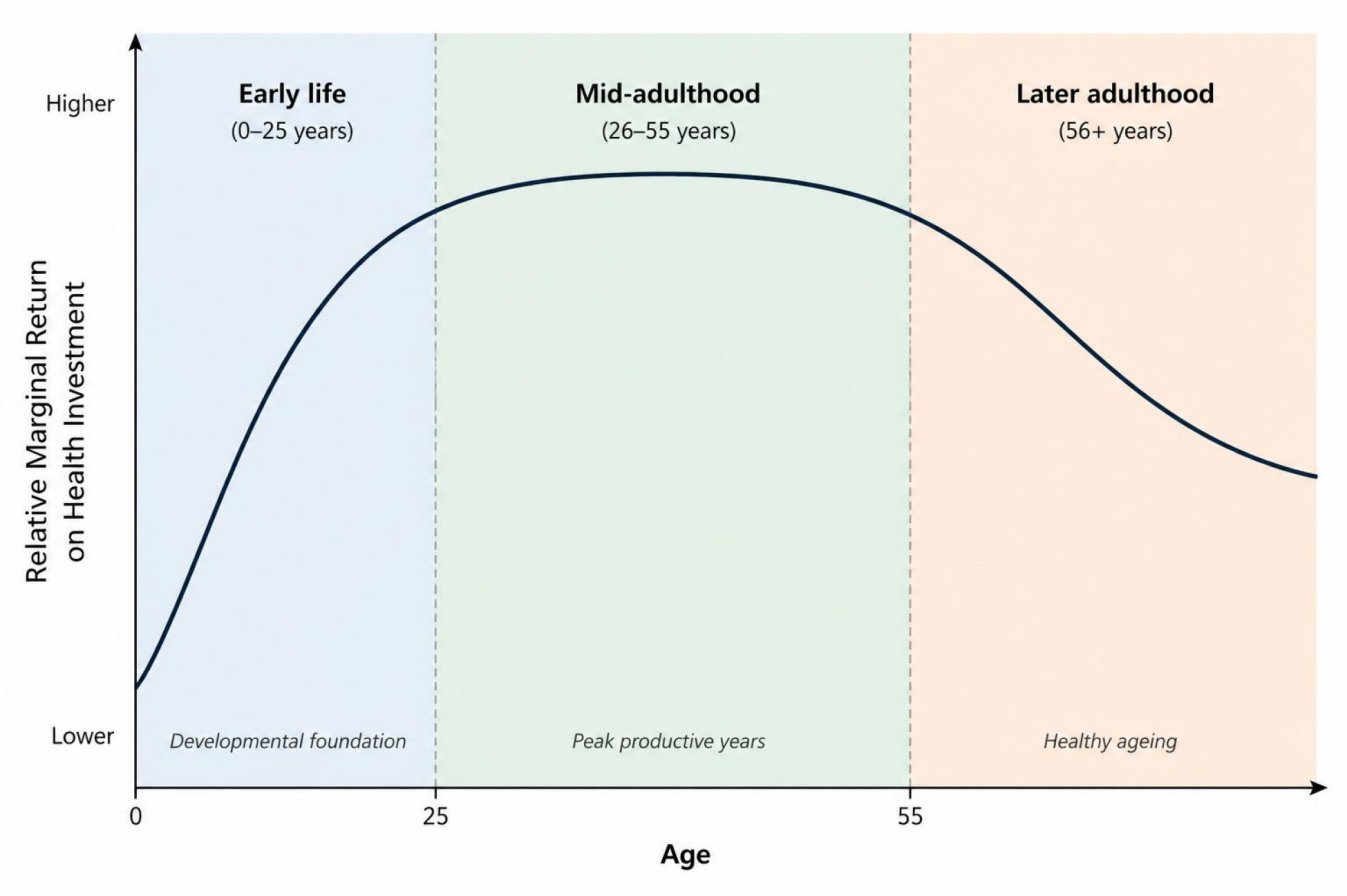
Conceptual framework of high-value age segments across the life course.

The proposed framework extends beyond policy approaches that focus primarily on preventing or delaying functional decline in later life, including the WHO Decade of Healthy Ageing (8). It places the timing of health investment at the centre of life-course resource allocation and asks where additional investment is likely to generate the greatest cumulative return. This logic is supported by evidence from developmental economics, health capital theory, and population health. Early-life interventions, including maternal and reproductive care, prenatal and neonatal screening, childhood vaccination, early nutrition, cognitive stimulation, and behavioural development, are linked to sustained gains in health, educational attainment, and later socioeconomic outcomes (11–14). Bloom et al. further show that healthier early-life cohorts generate measurable long-run productivity benefits (13). In mid-adulthood, cardiovascular risk reduction and chronic disease prevention can delay disease onset, preserve functional capacity, and reduce future healthcare expenditure. These patterns support age-stratified investment as a strategy for improving the long-term efficiency and sustainability of population health systems. Figure 1 presents this logic schematically. The curve is intended as an analytical organizer rather than a measured return distribution and is informed by Grossman's health capital model (10) and Heckman's analysis of declining returns to human capital investment with age (11).

The life course is divided into three segments: early life (fetal period to approximately 25 years), mid-adulthood (26–55 years), and later adulthood (56 years and above). The y-axis represents the relative return on health investment; the x-axis represents age. The curve is schematic and draws on Grossman's health capital model (10) and Heckman's analysis of declining returns to human capital investment with age (11), illustrating how marginal returns on health investment are expected to vary across broad life-course stages.

To translate this conceptual logic into an operational policy structure, Table 1 links each age segment to representative intervention priorities, supporting evidence, and anticipated population-level returns. The table is not intended as a quantitative ranking of age groups, but as an analytical guide for considering how different forms of health investment may generate distinct benefits across the life course.

**Table 1:** High-value age segments: intervention priorities, evidence base, and anticipated population-level returns.

Domain	Early life (0–25 years)	Mid-adulthood (26–55 years)	Later adulthood (56+ years)
Intervention focus	Maternal and reproductive care; prenatal and neonatal screening; childhood vaccination; early nutrition; cognitive and behavioural development	Cardiovascular risk reduction; chronic disease early detection; individual-level risk stratification; digitally enabled prevention	Integrated chronic disease management; geriatric care; rehabilitation; community-based support; morbidity compression strategies
Representative evidence base	Heckman (11); Jamison et al. (12); Bloom et al. (13); Smith et al. (16)	Topol (18); Awad et al. (19); Guo et al. (4)	WHO ICOPE (15); Gariboldi et al. (21); Horvath et al. (6)
Health returns	Reduced long-term disease risk; improved developmental health trajectories	Delayed disease onset; preserved functional capacity	Reduced morbidity burden; maintained intrinsic capacity
Functional and social returns	Improved educational attainment; strengthened human capital formation	Preserved workforce participation and productivity	Maintained independence and quality of life
System and economic returns	Long-run productivity gains; reduced future chronic disease burden	Reduced long-term healthcare expenditure	Reduced dependency burden and long-term care pressure

### 2.1 Operationalizing HVAS across the life course

The HVAS framework can be operationalized as a life-course strategy that links broad stages of life with different expected returns on health investment, while recognizing that health trajectories are cumulative and that investment at one stage influences outcomes at subsequent stages. Throughout this framework, “health investment” is used in a broad policy sense to refer to public resource allocation that generates population health returns, encompassing both health system expenditure and cross-sectoral investments in nutrition, early child development, and social protection. Three broad segments can be distinguished, each associated with distinct policy priorities and expected returns.

The early-life period, spanning the fetal stage to approximately 25 years, represents a critical window for establishing the biological and developmental foundations for later health and functioning (10, 11). Investment during this stage centres on foundational health and developmental processes, including maternal and reproductive care, prenatal and neonatal screening, childhood vaccination, early nutrition, and cognitive and behavioural development. These investments may influence biological resilience, educational attainment, and long-term disease risk, while contributing to broader human capital formation (11).

Mid-adulthood, approximately 26–55 years, corresponds to the period of peak physiological function and socioeconomic productivity. Health strategies during this stage emphasize targeted prevention, individual-level risk stratification, early chronic disease detection, and digitally supported follow-up. Maintaining health during this period may delay functional decline, sustain workforce participation, and reduce future demand for high-cost care.

Later adulthood, defined as 56 years and above, is characterized by increasing risk of multimorbidity and functional decline. The primary objective shifts toward morbidity compression through integrated chronic disease management, geriatric care, rehabilitation, and community-based support, with the aim of preserving intrinsic capacity and minimizing the duration of disability and dependency. Programs such as the WHO Integrated Care for Older People demonstrate that functional decline can be mitigated through coordinated interventions across health and social care systems (15). These age ranges are indicative rather than fixed and are intended to represent broad life-course stages relevant to health policy rather than precise biological or administrative boundaries.

### 2.2 Empirical validation and policy application of HVAS

For HVAS to function as a policy tool, its conceptual logic must translate into empirically testable propositions and practical allocation criteria. A natural question is whether life-course investment offers efficiency advantages over targeted preventive interventions directed at specific diseases or risk factors. These approaches are not mutually exclusive. The HVAS framework provides an age-stratified rationale for determining when targeted interventions are most likely to generate the greatest population-level returns. Disease-specific programs, such as neonatal metabolic screening in early life or cardiovascular risk assessment in mid-adulthood, derive much of their efficiency from being deployed during stages of high marginal return. Without an explicit life-course framework, targeted programs may be implemented in an *ad hoc* manner, with allocation shaped mainly by disease prevalence, institutional routines, or political salience rather than by expected long-term return on investment. A life-course approach therefore does not replace targeted prevention; it strengthens it by anchoring intervention packages to age segments where durable population health gains are most likely.

The theoretical plausibility of the HVAS framework is supported by the convergence of three evidence streams. Grossman's health capital theory establishes the economic logic of health as an investment good subject to accumulation and depreciation over time (10). Heckman's analysis of declining returns to human capital investment across the life course provides quantitative support for age-dependent differences in investment returns (11). The DOHaD literature further identifies biological mechanisms through which early-life exposures can produce lasting effects on disease risk and functional capacity. Together, these economic, developmental, and biological perspectives provide the conceptual foundation for HVAS. However, they do not substitute for direct empirical validation. Prospective testing should examine whether HVAS-defined age segments are associated with higher incremental gains in healthy life expectancy, disability-adjusted life-years averted, functional capacity, or long-term cost avoidance per unit of public expenditure.

### 2.3 Technological and system readiness for HVAS implementation

The feasibility of the HVAS framework is increasingly supported by advances in technology and health-system organization, although technology alone is insufficient for implementation. Across the life course, many of the technical prerequisites for age-stratified investment are already emerging. In early life, genomic technologies such as preimplantation genetic testing and non-invasive prenatal screening are widely implemented, and national newborn screening programs cover the majority of births in several countries (16). In mid-adulthood, digital health infrastructures that integrate electronic health records with artificial intelligence-assisted decision support can support large-scale risk stratification and preventive care, complemented by continuous monitoring technologies (17–19). In later life, long-term care systems and community-based integrated care models are expanding across the region, indicating that morbidity compression can be pursued through coordinated health and social care (20, 21). The principal constraint on HVAS implementation therefore lies less in technological feasibility than in the alignment of financing arrangements, governance structures, and population health priorities.

### 2.4 Policy implications and future directions

Operationalization of HVAS requires a gradual upstream rebalancing of health expenditure. Reallocation of resources toward earlier stages of the life course may mitigate future disease burden and associated costs without reducing investment in older populations. The HVAS framework also has an equity dimension: early-life interventions may generate particularly large marginal gains among children from socioeconomically disadvantaged backgrounds, where the benefits of timely investment are greatest and the costs of underinvestment most severe (11). Upstream prioritization may therefore help reduce the intergenerational transmission of health disadvantage, provided that access to HVAS-aligned interventions is equitably distributed across geographic and socioeconomic groups. Embedding HVAS within national planning frameworks, including China's 15th Five-Year Plan, Healthy China 2030, and WHO regional strategies, provides practical entry points for implementation (20, 22).

Equity safeguards should be built into any upstream investment strategy. These include geographic targeting in resource allocation, access provisions for rural and migrant populations, affordability protections for high-return preventive services, and routine monitoring of intervention access and health outcomes across socioeconomic and regional subgroups. Transparent monitoring and routine outcome reporting are necessary for accountability and for building the evidence base that HVAS-aligned systems will require. Methodological challenges remain, particularly in measuring life-course return on investment, because returns accrue across age stages over time horizons that typically exceed standard budgeting cycles.

## 3 Discussion

The Western Pacific Region is approaching a critical demographic inflection point. Continued prioritization of reactive, downstream care, together with persistent underallocation of public health resources to early-life and mid-life measures, including maternal and child health services, vaccination, childhood nutrition, early developmental support, and risk-based chronic disease prevention, is likely to reinforce a high-cost and low-efficiency equilibrium characterized by prolonged morbidity and increasing fiscal burden. The HVAS framework provides a structured and policy-relevant lens for rebalancing health investment across the life course through integration of economic evaluation and public health strategy.

Advancing HVAS from a conceptual framework to an evidence-based policy tool will require a structured empirical agenda. A foundational priority is developing life-course return-on-investment metrics capable of capturing health gains that accrue across age stages over time horizons extending well beyond conventional budgeting cycles. Standardized outcome indicators, including healthy life expectancy gained, disability-adjusted life-years averted, and long-term cost avoidance per unit of public expenditure stratified by HVAS-defined age segments, would enable cross-national comparison and policy benchmarking.

Equity-adjusted evaluation is equally necessary to determine whether HVAS-aligned investments narrow or widen health disparities across socioeconomic and geographic subgroups, particularly given that early-life returns are highest among disadvantaged populations (11). Implementation models will also need testing across diverse health system contexts using national health account datasets, longitudinal cohort studies, and WHO regional data infrastructures. Cross-national collaboration under WHO frameworks may support shared methodology and mutual learning.

The central contribution of HVAS is not to propose additional spending across all ages, but to make the timing of health investment an explicit dimension of resource allocation. The efficiency question facing aging societies is therefore not only which interventions to fund, but when public investment is most likely to compound into lasting population health gains. HVAS addresses this question by positioning investment timing as a core criterion for health resource allocation.

## Data Availability

The original contributions presented in the study are included in the article. Further inquiries can be directed to the corresponding author.
